# Outcome of Centenarians with Hip Fracture: An Analysis of the Registry for Geriatric Trauma (ATR-DGU)

**DOI:** 10.3390/jcm13216421

**Published:** 2024-10-26

**Authors:** Bastian Pass, Matthias Knobe, Hannah Schmidt, Christopher Bliemel, Rene Aigner, Ulrich Liener, Sven Lendemans, Carsten Schoeneberg, Ulf Boekeler, Registry for Geriatric Trauma (ATR-DGU)

**Affiliations:** 1 Department of Orthopedic and Emergency Surgery, Alfried Krupp Hospital, 45276 Essen, Germany; sven.lendemans@krupp-krankenhaus.de (S.L.); carsten.schoeneberg@krupp-krankenhaus.de (C.S.); 2 Department of Orthopaedic Trauma, Hospital Westmünsterland, 48683 Ahaus, Germany; matthias.knobe@kwml.de; 3 AUC—Academy for Trauma Surgery (AUC), 80538 Munich, Germany; hannah.schmidt@auc-online.de; 4 Center for Orthopedics and Trauma Surgery, University Hospital Giessen and Marburg, 35043 Marburg, Germany; bliemel@med.uni-marburg.de (C.B.); aignerr@med.uni-marburg.de (R.A.); 5 Department for Orthopaedics and Trauma Surgery, Marienhospital Stuttgart, Böheimstrasse 37, 70199 Stuttgart, Germany; ulrich.liener@vinzenz.de (U.L.); ulfwilhelm.boekeler@vinzenz.de (U.B.)

**Keywords:** centenarian, hip fracture, frailty, orthogeriatric, geriatric trauma

## Abstract

**Background/Objectives:** Outcomes for hip fracture patients have improved over the years, yet the population of older patients (≥80 years) continues to grow. By 2100, the global centenarian population is projected to exceed 25 million, but data on hip fracture outcomes in this group are rare and often derived from small samples. This study aimed to analyze outcomes for centenarian hip fracture patients in specialized geriatric trauma centers and compare them with those of patients under 80. **Methods:** We conducted a retrospective analysis of the AltersTraumaRegister DGU^®^ from 2016 to 2022, including all proximal femur fracture data. Patients were categorized into two groups: under 80 years and centenarians. The primary outcome was in-hospital mortality, with secondary outcomes including quality of life, walking ability on postoperative day seven, length of hospital stay, readmission rates, and changes in living situations. **Results:** Among 14,521 patients, 316 were over 99 years old. In-house mortality was significantly higher in centenarians (15.44% vs. 3.58%; *p* < 0.001), with more discharged to nursing homes. After matching by the Geriatrics at Risk (GeRi) score, mortality differences diminished. **Conclusions:** While age is a risk factor for mortality, centenarian hip fracture patients’ outcomes do not significantly differ from those aged ≤80 when considering other risk factors.

## 1. Introduction

Hip fractures are a leading cause of hospitalization among older patients. Due to demographic changes, a significant increase in the number of individuals (centenarians) who reach their 100th birthday has started to occur. Since 1950, the global number of centenarians has nearly doubled every decade [1,2]. Because of this increase, the global number of centenarians is predicted to reach more than 25 million by 2100 and the total number of hip fractures is projected to double in nearly all European countries up to 2050 [3]. About 40% of these patients will be older than 85 years [4]. The incidence of hip fractures increases exponentially with age, but data concerning very old patients are often pooled so that information about the incidence in over-100-year-olds is rare [4,5]. An observational study from the Spanish National Surveillance System for Hospital Data showed a hip fracture incidence of 3.8% in centenarians in 2005, and data from Denmark showed an incidence of 4.7% [6,7]. A recent meta-analysis stated that the current evidence base regarding outcomes in centenarians is of moderate to low quality [8]. Age is a known risk factor for early mortality in hip fracture patients and has become a parameter that has been incorporated into many mortality predictions models, such as the Geriatric at Risk Score (GeRi-Score) [9,10]. Besides medical issues, ethical dilemmas about the proper way of treating centenarians are also discussed [11]. On the other hand, centenarians are described as the lowest complexity patients with the lowest Charlson Co-morbidity Index (CCI) compared to younger hip fracture patients [12] and their comparatively healthy aging process is based on their genetic signature [13]. It is widely known that orthogeriatric care has led to a decrease in in-house mortality [14]. Relatively little is known about the oldest of the old despite their increasing numbers and the growing interest in this population. We conducted this study to analyze mortality in this remarkable population compared to the under 80-year-old patients group in certified centers for geriatric trauma. 

## 2. Materials and Methods

### 2.1. Data Sources

The ATR-DGU was established in 2016 by the Deutsche Gesellschaft für Unfallchirurgie (DGU). This multi-center database enables the pseudonymized and standardized documentation of patient information. To qualify for inclusion in the ATR-DGU, patients must be aged 70 years or older, diagnosed with a proximal femur fracture, and in need of surgical intervention. Patient data, which are anonymized, are submitted through a web-based platform into a central database by the participating hospitals prospectively. As of now, approximately 150 hospitals from Germany, Switzerland, and Austria contribute data, with around 60,000 cases recorded from 2016 to 2022. The scientific oversight of the registry is managed by the Working Committee on Geriatric Trauma Registry (AK ATR) within the DGU. Scientific analyses of the ATR-DGU data are authorized through a peer-review process, in line with the publication standards set by the AK ATR [15]. This study adheres to these publication guidelines and is registered under ATR-DGU project ID 2023-001.

### 2.2. Patients

The ATR-DGU inclusion criteria encompass patients with hip fractures, as well as those with periprosthetic and peri-implant fractures necessitating surgical intervention, specifically targeting individuals aged 70 years and older. We included all patients with a fracture of the hip who were older than 99 years and younger than 80 years. The analyzed data were obtained from specialized centers for geriatric trauma in Germany, Austria, and Switzerland. 

### 2.3. Covariates

Several variables were measured: (1) age, (2) sex, (3) American Society of Anesthesiologists (ASA) grade (from 1 to 5) [16], (4) the Identification of Seniors At Risk (ISAR) Score (from 0 to 6) [17], (5) the GeRi Score (Geriatrics at Risk Score) [9], (6) the use of anticoagulants (acetylsalicylic acid/aspirin was not considered an anticoagulant), (7) time to surgery, (8) current residential status, (9) walking ability before fracture, (10) on the seventh post-operative day, the presence of a geriatric before surgery, and (11) the presence of further injuries in addition to the hip fracture. Follow-up data were gathered on the 120th postoperative day. Quality of Life (QoL) was evaluated using the EuroQol quality of life instrument (EQ-5D-5L) on both the seventh and 120th postoperative days.

### 2.4. Outcomes

The primary outcome assessed was in-hospital mortality. Additional outcome measures included Quality of Life (QoL) evaluated through the EQ-5D-5L questionnaire, walking ability assessed on the seventh and 120th postoperative days, mortality at 120 days post-surgery, length of hospital stay (LoS), readmission rates, and any changes in living situations.

### 2.5. GeRi-Score

The GeRi-Score was created using data from the Registry for Geriatric Trauma to predict in-hospital mortality in geriatric patients with hip fractures. The model incorporates several covariates, including: (1) ASA grade (1 to 5), (2) anticoagulant use, (3) sex, (4) age, (5) types and presence of concomitant injuries, and (6) the location of the patient’s fall. The GeRi-Score ranges from 0 to 20 points, calculated by summing the scores of all covariates based on individual patient characteristics [9].

### 2.6. Statistical analyses

All statistical analyses were conducted using statistics software R version 4.1.2 (Foundation for Statistical Computing, Vienna, Austria). For descriptive analyses, categorical data were presented as numbers and percentages and continuous variables, such as median with interquartile range (IQR). Due to some missing data for specific parameters, the analyses reflect the total number of patients included in each assessment. A 2:1 nearest neighbor matching based on the GeRi-Score was applied. The results from the EQ-5D-5L questionnaires were converted into a single index utilizing the German (GER) Ludwig value set, Version 2.1 [18]. For patients with documented EQ-5D-3L questionnaire results, cross walk mapping was performed to obtain the EQ-5D-5L index value, which ranges from −0.661 (indicating the worst health status) to 1 (indicating the best health status). Comparisons between groups (<80 years versus >99 years) were obtained using the chi-squared test for categorical variables and the Wilcoxon test for continuous variables. Linear and logistic regression models were used to examine the impact of age on outcomes after controlling for ASA grade, sex, anticoagulants at admission, type of proximal femur fracture, and presence of injuries other than the hip fracture. Results were reported as regression coefficients (b) for linear regression and odds ratios for logistic regression along with their 95% confidence intervals. Differences were considered statistically significant when *p* < 0.05.

### 2.7. Ethics

Written informed consent was obtained from patients by the participating hospitals. The data from the ATR-DGU received full approval from the Ethics Committee of the Medical Faculty at Philipps University Marburg, Germany (AZ 46/16), on 5 April 2018.

## 3. Results

**Figure 1:**
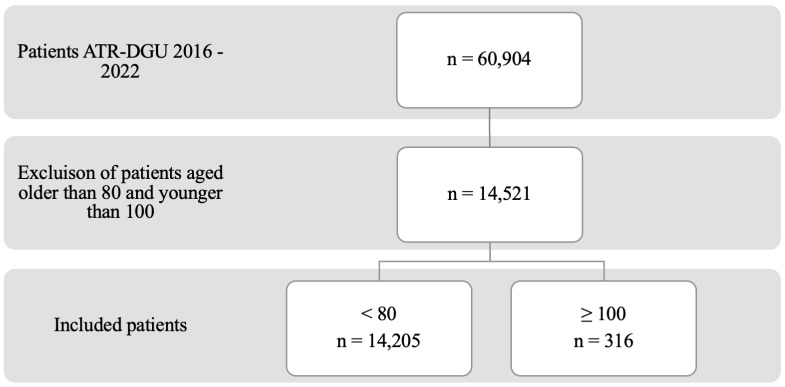
Flow sheet for patient inclusion.

After excluding patients younger than 100 and older than 79, a total of 14,521 patients remained in the study cohort. Within this cohort, 316 patients were over 99 years of age (Figure 1). Notably, patients in this age group were more likely to be female and displayed a higher number of comorbidities, as indicated by their ASA grade. Additionally, these patients had elevated ISAR scores, took fewer anticoagulants, and showed diminished independent walking capabilities. They were also more likely to reside in nursing homes. No significant differences were observed regarding concomitant injuries or pre-surgery geriatric visits between the groups. The baseline data are summarized in Table 1.

### 3.1. Outcome Before Matching

Nearly every second patient over 100 years old lived in a nursing home, and only 7.48% were independently walking before the fracture. In the centenarian group, surgeries were performed earlier compared to the under-80 group, and these patients were more frequently permitted to bear full weight post-surgery. Quality of Life (QoL) scores were lower on the seventh post-surgical day and nearly half of the centenarians showed no walking ability at that time. However, the older group demonstrated a greater improvement in walking ability, with an increase of 21.03% compared to 8.77% in the younger cohort (*p* < 0.001). In-house mortality rates were significantly higher among centenarians, at 15.44% versus 3.58% in younger patients (*p* < 0.001). Notably, for centenarians who lived independently before their injury, nearly a third were discharged to nursing homes, compared to just 8.66% in the under-80 group. Data from 5413 patients were available at the 120-day follow-up mark. During this period, one in three patients aged over 99 years passed away. Table 2 presents detailed information on inpatient outcomes and follow-ups.

### 3.2. Outcome after Matching Based on the GeRi-Score

Due to significant differences in baseline characteristics, nearest neighbor matching was applied using the GeRi-Score. Following this process, 586 patients from the younger cohort and 293 centenarians remained. Although centenarians exhibited significantly lower capabilities for independent walking, Quality of Life (QoL) scores did not differ between the two groups on the seventh post-surgical day, and in-house mortality rates showed no significant differences. Notably, nearly one in three centenarians who lived independently before the fracture were discharged to nursing homes post-surgery (Table 3).

## 4. Discussion

Our results demonstrate that centenarians are at high risk for in-house mortality and discharge to a nursing home after hip fracture surgery. We specifically compared the mortality rates of hip fracture patients in two distinct age groups: those under 80 years old and those over 100 years old. This comparison allows us to assess the centenarians against a geriatric group expected to have the highest survival rates, as age is a well-known risk factor for mortality [9,10].

The in-house mortality rate in the centenarian group was 15%, which was about four times higher compared to the under 80s. The results for patients under 80 years old are consistent with recent literature [19]. Our analyzed data were obtained from specialized centers for geriatric trauma in Germany, Austria, and Switzerland. Data from the UK showed an in-house mortality of 13% in centenarians [20] while Spanish data came up with a mortality of 33% [21]. A systematic review and meta-analysis with data from 16 studies consisting of 4464 patients showed a mortality rate of 16% for those who underwent surgery for hip fractures [8]. 

Any advantage or disadvantage of a conservative procedure in terms of mortality in older patients has not been demonstrated so far [8]. However, data from conservatively treated nonagenarians suggest that the non-operative group may experience higher mortality rates. At such an advanced age, surgical interventions often assume a more palliative role, primarily focusing on pain relief and improving quality of life rather than curative intent. This highlights the need for a tailored approach to treatment, considering the specific health profiles and needs of older patients in clinical decision-making [20,22]. 

The ASA grade has shown a correlation with mortality in hip fracture patients [23]. In our analysis, the centenarians had a significantly higher ASA grade since nearly 90% achieved a grade of 3 or above 3, whereas the grade was less than 3 in 70% in the under 80-year-old patients. Using the ASA grade as a surrogate for comorbidities, it appears likely that mortality increases in correspondence with higher ASA grades, as shown in the development of the GeRi-Score. Therefore, it is noteworthy that other studies have shown that centenarians exhibited a lower Charlson Comorbidity Index—another surrogate parameter for comorbidities—which also predicts mortality similarly to the ASA grade. Additionally, centenarians tend to have a lower daily drug intake compared to the younger cohort. [7,8,12,23]. 

When considering time-to-surgery as a parameter for anesthetic risk assessment, we found that the older patient group was treated significantly earlier, despite being more likely to live in a nursing home and having significantly higher ASA grades and ISAR scores, which indicate a greater risk of mortality [17,24]. On the other side the older patients had a lower intake of anticoagulants, which could accelerate the time-to-surgery [25]. However, the optimal time-to-surgery concerning mortality and complications remains a topic of debate [26,27,28].

To reduce mortality in geriatric hip fracture patients, orthogeriatric care was successfully established [14,29,30]. Although great efforts have been achieved with orthogeriatric co-management, the number of patients, who were living in their own home before surgery, discharged to a nursing home after hip fracture surgery was significantly higher in the older group. Since we did not observe any differences in the preoperative geriatric visit, we assume that the potential for rehabilitation was regarded as lower than in the younger cohort. Fewer patients were discharged to another inpatient facility for rehabilitation (58.09% vs. 35.38%, *p* < 0.001).

A comparison of mortality with the under-80s using the GeRi score, a mortality predictor for patients with hip fractures, was conducted. We chose the GeRi-Score as this a tool for predicting mortality, demonstrating acceptable discrimination without significant lack of fit and all the needed data can be found in the AltersTraumaRegister DGU^®^. The GeRi-Score was selected for this comparison because it effectively accounts for diverse risk profiles, combining age and significant comorbidities. This scoring system allows for a nuanced understanding of mortality risk, acknowledging that age alone does not fully capture the complexities involved. By using the GeRi-Score, we can predict mortality based on various items, facilitating a more accurate comparison between younger patients with comorbidities and centenarians, who inherently face higher risks due to their age [9].

It was found that age alone is not sufficient to account for an increase in mortality. After matching on the basis of the GeRi score, we found no significant differences in inpatient mortality between the two groups.

However, other parameters, such as time-to-surgery and discharge targets, remained significantly different. Many factors can influence time-to-surgery, including the use of anticoagulants, which must be considered when assessing the results. Patients were matched on their GeRi score, a single value summarizing various risk factors, but not directly on one specific risk factors, like anticoagulants or gender.

The previous observed trend of the discharged remained the same after matching for GeRi-Score. Almost every third centenarian who lived in his own home prior to the fracture was discharged to a nursing home. This finding might reflect the high proportion of poor post-surgical walking ability. It can be assumed here that the clinical picture of sarcopenia, as an age-related form of muscle mass loss, certainly plays an important role. Similar discharge proportions could be observed in the study by Oliver and Burke, who also revealed that only 22% of the centenarians regained their pre-fracture walking ability [31]. Thirty-six percent of our included centenarians had no change in walking ability, and 21% showed improvements in walking ability, whereas nearly 70% of the under 80-year-old patients presented worse walking ability.

It is noteworthy that a larger proportion of older patients showed improved walking ability compared to the younger cohort. This observation raises interesting discussions about the perceptions of surgical intervention among older individuals. Many patients may fear the risks associated with elective surgery for osteoarthritis, particularly at such an advanced age, leading them to avoid surgery until absolutely necessary. After a fracture occurs, these patients often find themselves with limited options, resulting in a lack of choice regarding surgical intervention. 

Nevertheless, the majority in both groups are losing their pre-fracture walking capability. This may partially explain the low EQ-5D scores observed in both groups on the seventh day after surgery. This assessment is likely to change once patients regain mobility and their ability for self-care following rehabilitation. However, we do not have sufficient data on the quality of life at day 120 after surgery in the centenarian group to make a definitive statement. Data on the quality of life in centenarians is relatively scarce. Cross-sectional data from China revealed an EQ-5D index of 0.62 in centenarians [32]. Data regarding the quality of life, as measured by the EQ-5D, in the general centenarian population from Germany, Switzerland, and Austria are not available in the literature. 

## 5. Conclusions

This study shows that age alone is not the decisive parameter affecting older patient in-house mortality and functional status. Although centenarians present a select group of individuals with a probably healthier constitution [33] they still possess a high propensity of frailty. Therefore, research with this special subgroup of patients may lead to a better understanding and treatment of geriatric hip fracture patients. Furthermore, future studies should examine the influence of geriatricians on patient outcomes. A risk assessment, like the GeRi-Score, can assist in directing geriatricians to patients who need their specialized expertise.

## 6. Limitations

This study has several limitations that must be acknowledged. Firstly, it was conducted as a retrospective analysis, which may introduce potential biases such as selection bias and confounding. However, it is noteworthy that the data for the ATR-DGU were collected prospectively, which helps mitigate some of these concerns.

It is essential to recognize that the reliability of registries is contingent upon the accurate collection and entry of data. To uphold data integrity, a certification process and regular audits are conducted for all hospitals involved in the ATR-DGU.

Another limitation of this study is that follow-up assessments at 120 days post-surgery are optional for patients, resulting in a smaller sample size and the potential for selection bias. This may affect the generalizability of the findings. Additionally, while the data are sourced from specialized geriatric trauma centers, the quality of treatment in these facilities may not reflect the standards of care provided across all hospitals. Consequently, the outcomes observed in this study might not be representative of the broader healthcare setting.

## Figures and Tables

**Table 1:** Comparison of the baseline data.

	<80 Years	>99 Years	*p*-Value
Patients	n = 14,205	n = 316	
Age [Years]	n = 14,205	n = 316	
Median (IQR)	76 (73–78)	101 (100–102)	
Sex	n = 14,181	n = 316	<0.001 *
Male	4960 (34.98%)	39 (12.34%)	
Female	9221 (65.02%)	277 (87.66%)	
Type of fracture	n = 14.138	n = 312	<0.001 *
Femoral neck	6927 (49%)	109 (34.94%)	
Per-/Subtrochanteric fracture	6051 (42.8%)	184 (58.98%)	
Periprosthetic fracture	757 (5.35%)	10 (3.21%)	
Other	403 (2.85%)	9 (2.88%)	
Pathologic fractures	n = 12,993	n = 289	0.033 *
Atypical fracture	74 (0.57%)	0 (0%)	
Cancer	193 (1.49%)	0 (0%)	
ASA grade	n = 13,862	n = 304	
Median (IQR)	3 (2–3)	3 (3–3)	<0.001 *
1	302 (2.18%)	0 (0%)	
2	4219 (30.44%)	40 (13.16%)	
3	8436 (60.86%)	220 (72.37%)	<0.001 *
4	894 (6.45%)	43 (14.14%)	
5	11 (0.08%)	1 (0.33%)	
ISAR-Score	n = 10,556	n = 235	
Median (IQR)	2 (1–3)	3 (2–4)	<0.001 *
0	1768 (16.75%)	3 (1.28%)	
1	1818 (17.22%)	12 (5.11%)	
2	2353 (22.29%)	45 (19.15%)	
3	2147 (20.34%)	72 (30.64%)	<0.001 *
4	1683 (15.94%)	70 (29.79%)	
5	634 (6.01%)	25 (10.64%)	
6	153 (1.45%)	8 (3.4%)	
Anticoagulation	n = 13,678	n = 309	<0.001 *
Yes	6402 (46.81%)	112 (36.25%)	
Living situation before fracture	n = 13,932	n = 313	<0.001 *
At home	12,065 (86.6%)	159 (50.8%)	
Nursing home	1584 (11.37%)	151 (48.24%)	
Hospital	177 (1.27%)	2 (0.64%)	
other	106 (0.76%)	1 (0.32%)	
Walking ability before injury	n = 13,240	n = 294	<0.001 *
Independent without walking frame	7147 (53.98%)	22 (7.48%)	
Out of house walking with one crutch	1300 (9.82%)	20 (6.8%)	
Out of house walking with two crutches or other walking frame	3082 (23.28%)	104 (35.37%)	
Walking ability within apartment, outside only with auxiliary person	1389 (10.49%)	118 (40.14%)	
No functional walking ability	322 (2.43%)	30 (10.2%)	
Concomitant injury	n = 14,152	n = 315	0.595
Yes	483 (3.41%)	13 (4.13%)	
Pre-surgery geriatric visit	n = 10,345	n = 233	0.316
Yes	2268 (21.92%)	58 (24.89%)	

The number of patients with data is given separately for each parameter. ASA, American Society of Anesthesiologists; ISAR, Identification-of-Seniors-At-Risk; IQR, interquartile range, * significant differences.

**Table 2:** Differences in inpatients’ outcome.

	<80 Years	>99 Years	*p*-Value
Patients	n = 14,205	n = 316	
Time-to-surgery [hours]	n = 14,057	n = 311	
Median (IQR)	17 (7–24)	15 (7–22)	<0.032 *
Length of hospital stay for surviving patients [days]	n = 12,708	n = 251	
Median (IQR)	14 (9–21)	15 (8–20)	0.168
Full weight bearing	n = 14,099	n = 314	
Yes	12,390 (87.88%)	290 (92.36%)	0.02 *
Type of anaesthesia **	n = 14,151	n = 314	
General anaesthesia	13,039 (92.14%)	269 (85.67%)	
Spinal	1009 (7.18%)	38 (12.1%)	
Other	378 (2.67%)	19 (6.05%)	
EQ-5D-5L 7 days after surgery	n = 9956	n = 182	
Median (IQR)	0.62 (0.45–0.75)	0.41 (0.24–0.62)	<0.001 *
Walking ability on the 7th day after surgery	n = 13,559	n = 290	
Without walking aids	174 (1.28%)	2 (0.69%)	<0.001 *
With crutches	2900 (21.39%)	5 (1,72%)	
With rollator	3716 (27.41%)	57 (19.66%)	
With wheeled walker	2840 (20.95%)	63 (21.72%)	
With walking frame	1699 (12.53%)	35 (12.07%)	
Not possible	2230 (16.45%)	128 (44.14%)	
Change in walking ability	n = 12,726	n = 271	
No change	2779 (21,84%)	99 (36.53%)	<0.001 *
Better	1116 (8.77%)	57 (21.03%)	
Worse	8831 (69.39%)	115 (42.44%)	
Geriatric Visit	n = 13,832	n = 301	0.592
Yes	11,345 (82.02%)	251 (83.39%)	
Died during hospital stay	n = 13,274	n = 298	
Yes	475 (3.58%)	46 (15.44%)	<0.001 *
Re-surgery during inpatient stay	n = 14,193	n = 316	
Yes	400 (2.82%)	7 (2.22%)	0.638
Discharge target for patients living in their own home before injury	n = 11,564	n = 136	
Own house	4496 (38.88%)	44 (32.35%)	<0.001 *
Nursing home	1001 (8.66%)	41 (30.15%)	
Inpatient facility	6067 (52.46%)	51 (37.5%)	
Died during follow up	n = 4599	n = 77	
Yes	285 (6.2%)	26 (33.77%)	<0.001 *

The number of patients with data is given separately for each parameter. * significant differences; ** multiple choice outcome.

**Table 3:** After 2:1 matching on GeRi Score.

	<80 Years	>99 Years	*p*-Value
Patients	n = 586	n = 293	
GeRi Score			
Median (IQR)	8 (7–9)	8 (7–9)	
Age [Years]	n = 586	n = 293	
Median (IQR)	77 (74–78)	101 (100–102)	
ASA grade	n = 586	n = 293	
Median (IQR)	3 (3–4)	3 (3–3)	<0.001 *
1	0 (0%)	0 (0%)	<0.001 *
2	4 (0.68%)	37 (12.63%)	
3	335 (57.17%)	212 (72.35%)	
4	242 (41.30%)	43 (14.68%)	
5	5 (0.85%)	1 (0.34%)	
ISAR-Score	n = 441	n = 218	
Median (IQR)	3 (2–4)	3 (2–4)	0.711
0	16 (3.63%)	0 (1.38%)	0.011
1	40 (9.07%)	11 (5.05%)	
2	68 (15.42%)	41 (18.81%)	
3	105 (23.81%)	68 (31.19%)	
4	138 (31.29%)	63 (28.90%)	
5	54 (12.24%)	24 (11.01%)	
6	20 (4.54%)	8 (3.67%)	
Sex	n = 586	n = 293	<0.001 *
Male	484 (82.59%)	37 (12.63%)	
Female	102 (17.41%)	256 (87.37%)	
Anticoagulation	n = 586	n = 293	<0.001 *
Yes	445 (75.94%)	105 (35.84%)	
Concomitant injury	n = 14,152	n = 293	0.001
Yes	57 (9.73%)	10 (3.41%)	
Pre-operative geriatric visit	n = 381	n = 216	0.604
Yes	85 (22.31%)	53 (24.54%)	
Time-to-surgery [hours]	n = 580	n = 289	
Median (IQR)	19 (7–32)	15 (7–22)	<0.001 *
Length of hospital stay for surviving patients [days]	n = 475	n = 234	
Median (IQR)	15 (10–22)	15 (8–21)	0.03 *
Full weight bearing	n = 586	n = 291	
Yes	528 (90.10%)	271 (93.13%)	0.175
Type of anaesthesia **	n = 585	n = 285	
General anaesthesia	552 (94.36%)	247 (84.87%)	
Spinal	31 (5.3%)	38 (9.62%)	
Other	13 (2.22%)	18 (6.18%)	
EQ-5D-5L 7 days after surgery	n = 362	n = 172	
Median (IQR)	0.505 (0.261–0.687)	0.412 (0.233–0.62)	0.11
Walking ability on the 7th day after surgery	n = 557	n = 270	
Without walking aids	2 (0.36%)	2 (0.74%)	<0.001 *
With crutches	30 (5.39%)	5 (1.85%)	
With rollator	113 (20.29%)	54 (20.00%)	
With wheeled walker	145 (26.03%)	60 (22.22%)	
With walking frame	72 (12.93%)	33 (12.22%)	
Not possible	195 (35.01%)	116 (42.96%)	
Change in walking ability	n = 509	n = 253	
No change	131 (25.74%)	92 (36.36%)	<0.001 *
Better	78 (15.32%)	55 (21.74%)	
Worse	300 (58.94%)	106 (41.90%)	
Geriatric visit	n = 579	n = 291	
Yes	456 (78.76%)	235 (83.63%)	0.111
Died during hospital stay	n = 539	n = 277	
Yes	61 (11.32%)	42 (15.16%)	0.146
Re-surgery during inpatient stay	n = 586	n = 293	
Yes	25 (4.27%)	7 (2.39%)	0.226
Discharge target for patients living in their own home before injury	n = 303	n = 130	
Own house	89 (29.37%)	44 (33.85%)	<0.001 *
Nursing home	38 (12.54%)	40 (30.77%)	
Inpatient facility	176 (58.09%)	46 (35.38%)	
Died during follow-up	n = 182	n = 76	
Yes	27 (14.84%)	25 (32.89%)	0.002 *

The number of patients with data is given separately for each parameter. * significant differences; ** multiple choice outcome.

## Data Availability

Restrictions apply to the availability of these data. Data were obtained from the Registry for Geriatric Trauma (ATR-DGU) and are available from the Academy for Trauma Surgery (AUC) with the permission of the Working Committee on Geriatric Trauma Registry (AK ATR) of the German Trauma Society (DGU).
